# Pancreatic solitary metastasis of lung squamous cell carcinoma: A rare case report

**DOI:** 10.1097/MD.0000000000047234

**Published:** 2026-01-16

**Authors:** Congying Hu, Zhirong Shen, Hongen Zhen, Dongmei Rao, Xuejin Sun, Jianqun Yu

**Affiliations:** a Department of Medical Imaging, Northeast Yunnan Central Hospital, Zhaotong, Yunnan Province, China; b Department of Radiology, Kunming Municipal Hospital of Traditional Chinese Medicine, Kunming, Yunnan Province, China; c Department of Radiology, West China Hospital of Sichuan University, Chengdu, Sichuan Province, China.

**Keywords:** lung squamous cell carcinoma, pancreatic metastasis, radical resection

## Abstract

**Rationale:**

Lung cancer is a common malignancy worldwide, but its metastasis to the pancreas is rare. Among lung cancer cases with pancreatic metastasis, small cell carcinoma is one of the most common pathological types, while squamous cell carcinoma is a very rare type. Most patients with pancreatic metastases are asymptomatic, which can easily lead to missed diagnosis.

**Patient concerns:**

The patient was a 61-year-old man who presented with a solitary mass in the pancreatic tail over 20 months after undergoing surgery for squamous cell carcinoma of the right lower lung. A retrospective review of imaging studies revealed that pancreatic lesions had been missed on contrast-enhanced abdominal computed tomography scans performed at 14 and 16 months after the lung surgery. The patient subsequently underwent radical surgical resection of the pancreatic metastases, which confirmed the diagnosis of metastatic squamous cell carcinoma.

**Diagnoses:**

A pancreatic mass was identified on contrast-enhanced abdominal computed tomography and magnetic resonance imaging. Pathological examination of the resected specimen confirmed metastatic squamous cell carcinoma of lung origin.

**Interventions:**

Radical surgical resection.

**Outcomes:**

A solitary mass was discovered in the pancreas and was pathologically confirmed to be a metastasis from the patient’s primary squamous cell carcinoma of the right lower lung, which had been resected 20 months prior. The patient died from progression of the metastatic disease 10 months after undergoing a right nephrectomy.

**Lessons:**

Pancreatic metastasis of lung squamous carcinoma is rare and insidious. Therefore, radiologists should meticulously review imaging studies to detect smaller lesions in a timely manner. This vigilant approach is crucial for ensuring patients receive appropriate treatment and potentially prolonging their survival.

## 1. Introduction

Lung cancer is the leading cause of cancer-related deaths globally.^[1]^ Research indicates that only 1% of non-small cell lung cancers metastasize to the pancreas.^[2]^ Among pancreatic metastases, pulmonary squamous cell carcinoma is the rarest pathological type in pancreatic metastasis from lung cancer,^[3-5]^ accounting for just 1.1%.^[6]^ Most patients with pancreatic metastases present with vague symptoms and nonspecific signs, which can lead to missed diagnoses.

Patients with pancreatic metastasis from lung cancer have a poor prognosis, with the longest median survival being 29 months.^[7,8]^ Prognosis depends not only on the stage of the primary lung cancer and the treatment plan but also on the patient’s neutrophil-to-eosinophil ratio (NER),^[9]^ cachexia index (CXI),^[10]^ and other comorbidities like type 2 diabetes, metabolic syndrome, and obesity, which are linked to cancer progression.^[11]^

Solitary pancreatic metastasis is a rare manifestation of primary lung cancer and is associated with an unfavorable prognosis.^[12]^ Given the limited reports on pulmonary squamous cell carcinoma metastasizing to the pancreas, the authors present a case of a patient who developed a solitary pancreatic tail metastasis 14 months after radical lung cancer surgery. This case stresses the need for radiologists to thoroughly examine images to detect small or occult lesions and reduce missed diagnoses. It also serves as a reminder for clinicians to assess patients’ comorbidities, NER, and CXI to identify high-risk patients, evaluate prognosis, adjust treatment strategies, and potentially extend survival.

## 2. Case presentation

The patient, a 61-year-old male, was admitted to our hospital on November 3, 2021, because of “a solitary lesion at the tail of the pancreas was reported for 1+ month, which occurred 20 months after the operation of lung squamous cell carcinoma in the lower right lung.”

**Figure 1.**
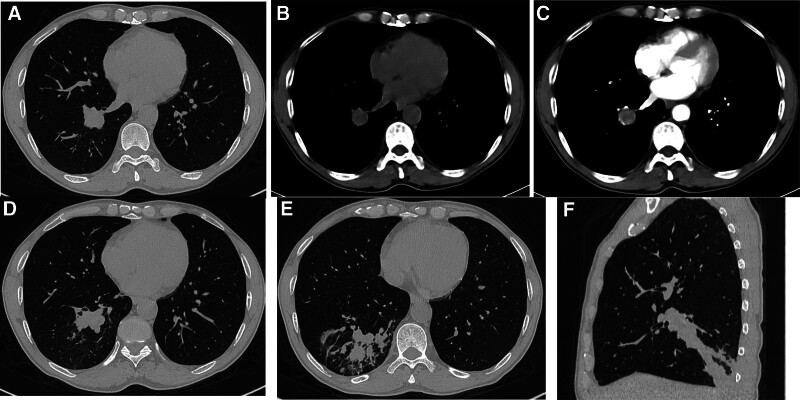
Images of on January 6, 2020 enhanced-chest CT, demonstrating mass shadow was observed in the basal segment of the lower lobe of the right lung (A, B), uneven enhancement on enhanced scanning (C), local bronchial stenosis and occlusion, multiple solid shadows, patches and cable shadows in the distal lung field (D–F). CT = computed tomography.

On January 6, 2020, the patient developed repeated irritant cough and blood in sputum without obvious inductions, with intermittent symptoms. Chest computed tomography (CT) scan at a local hospital revealed infectious lesions in the lower lobe of the right lung. Anti-infection treatment (specific details are unknown) proved ineffective. For further diagnosis and treatment, the patient was admitted to our hospital and received chest enhanced CT examination (Fig. 1): mass shadow was observed in the basal segment of the lower lobe of the right lung, distributed along the bronchial vascular bundle, with blurred boundaries, uneven enhancement by enhanced scanning, local bronchial stenosis and occlusion, multiple solid shadows, patches and cable shadows in the distal lung field. The right hilar and mediastinal lymph nodes were slightly enlarged. Imaging diagnosis: Neoplastic lesions are more likely to be combined with obstructive pneumonia. On February 3, 2020, serum tumor markers were examined: carcinoembryonic antigen, cytokertin 19, and neuron-specific enolase were all within the normal range. The chest CT examination in the same month showed an enlargement of the lesion in the lower lobe of the right lung. At the same time, bronchofibroscopy found that the opening of the anterior, outer and posterior basal segments bronchi of the lower lobe of the right lung was blocked by cauliflower-like new organisms. Histopathologic examination revealed squamous cell carcinoma. Then head enhanced magnetic resonance imaging (MRI), whole abdomen enhanced CT and total body bone scan showed no definite signs of distant metastasis. In the same month, the patient underwent the lower lobe resection of right lung via right thoracotomy and systemic lymph node dissection under general anesthesia. The postoperative pathology diagnosis was keratinized squamous cell carcinoma in the lower lobe of the right lung, moderately differentiated. Immunohistochemistry showed TTF-1(−), CK5/6(+), P63(+), P40(+), PCK(+). Targeted detection: ALK-V(−), ROS-1(−); no carcinoma involvement in bronchial stump; no vascular cancer thrombus; some of the hilar and mediastinal lymph nodes were metastasized. The postoperative pathological stage was T1N2M0 IIIA.

**Figure 2.**
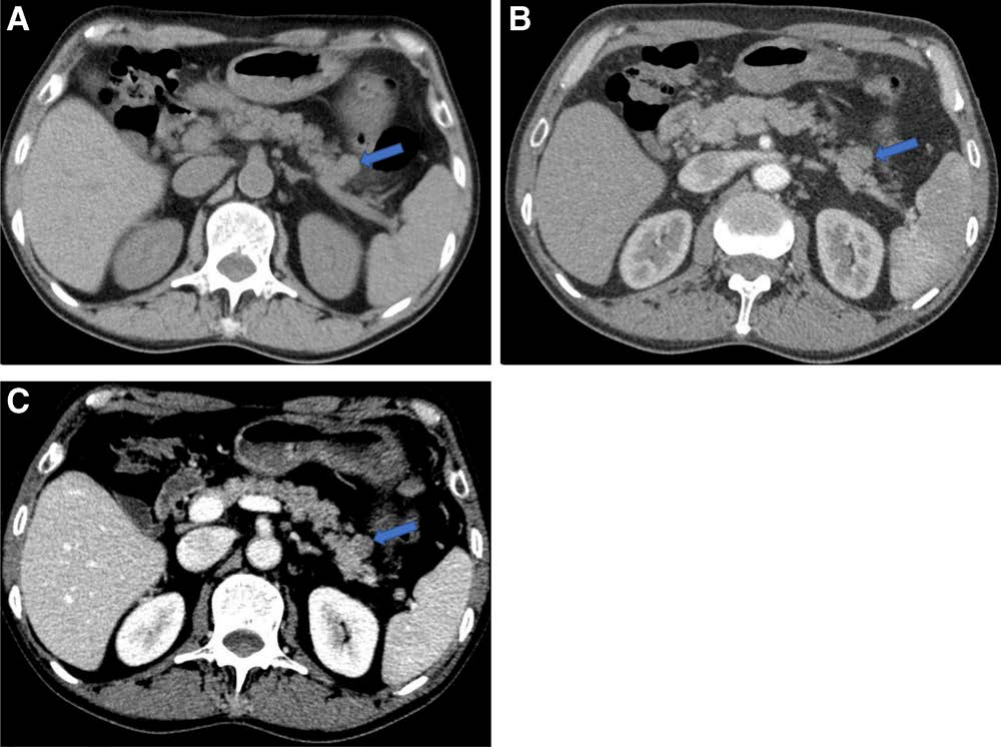
May 24, 2021 (14+ months after lung cancer surgery) enhanced abdominal CT examination. Plain scan (A) showed first order density nodule (blue arrow) in the tail of pancreas; arterial and venous phases (B, C) showed slight enhancement of the nodule of the tail of the pancreas, which was weaker than that of the adjacent pancreatic parenchyma, and the boundary was clearer than that of the plain scan. CT = computed tomography.

**Figure 3.**
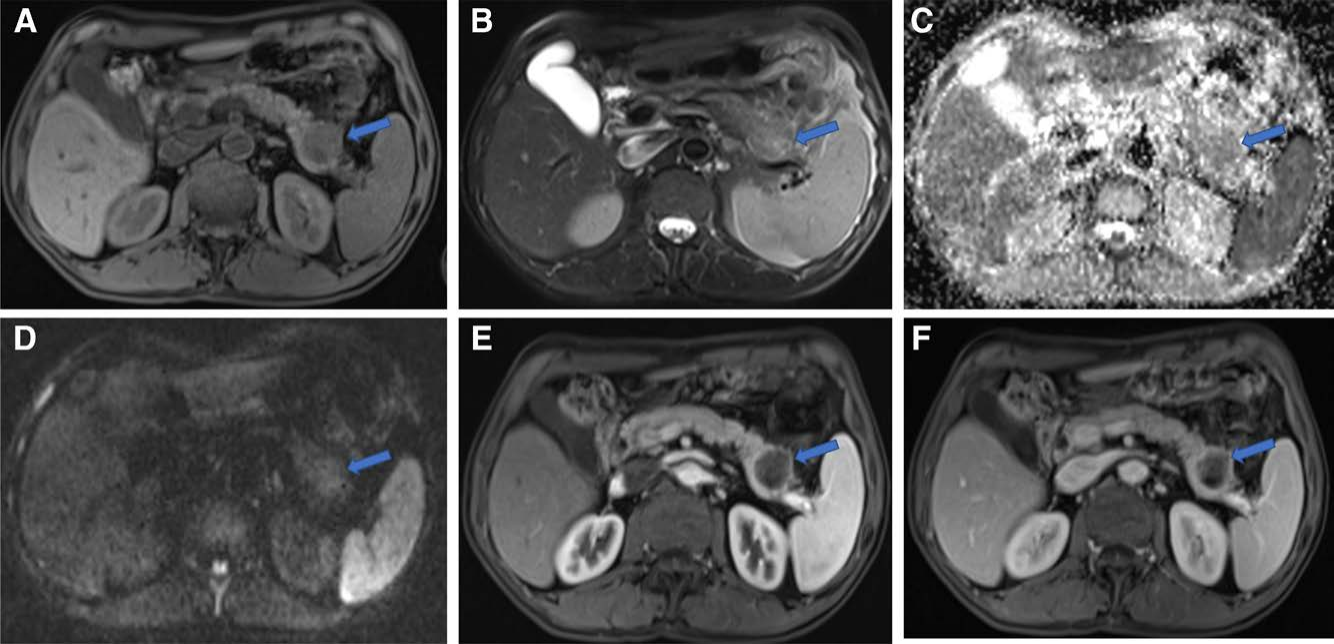
October 22, 2021 upper abdominal contrast-MRI, demonstrating the lesion in the tail of the pancreas, with 3.1 cm × 2.9 cm mass, slightly low signal on T1WI (A), mixed slightly high signal on T2WI (B), and had clear boundary, mild limited diffusion, the mean ADC value is approximately 1154 × 10⁻³ mm²/s (C, D), and rim enhancement on enhanced images (E, F). ADC = apparent diffusion coefficient, MRI = magnetic resonance imaging.

The patient underwent 4 cycles of paclitaxel liposome 220 mg D1+ cisplatin 40 mg D1–D3 chemotherapy after the operation of lung cancer, and was regularly reexamined with fiberoptic bronchoscopy, chest and abdomen enhanced CT scan and head enhanced MRI scan. No signs of recurrence or metastasis were found within the first year after the lung cancer operation. However, 12 months after surgery, bronchofibroscopy revealed nodules at the bronchial stump of the right lower lobe. Biopsy pathology confirmed squamous cell carcinoma (keratinizing type), which was interpreted as tumor recurrence. At the same time, chest CT examination showed that soft tissue mass shadow located in the bronchial stump of in the lower lobe of the right lung and was considered to be tumor recurrence, with 3.4 cm × 2.2 cm in size. Whole abdominal enhanced CT and head enhanced MRI scan showed no definite signs of metastasis. After that, post-relapse chemotherapy was given, specifically albumin paclitaxel 450 mg, D1+ carboplatin 480 mg, D1+ carrilizumab 200 mg D1, q3w. Abdomen-pelvis enhanced CT scans reported no exact metastatic lesions in the abdomen (retrospective review of the radiographs observed a oval iso-dense nodule in the tail of the pancreas at the time of 14 and 16 months after lung cancer surgery, with sizes about 1.3 cm × 1.1 cm (Fig. 2) and 1.9 cm × 1.4 cm, respectively, with clear boundaries and mild enhancement). Serum tumor markers were all within the normal range. After 6 cycles of chemotherapy, enhanced CT scans in the chest and abdomen showed that there were a few soft tissue shadows in the bronchial stump of the lower lobe of the right lung. The size of the nodule in the tail of the pancreas was about 2.6 cm × 2.4 cm, which was larger than before, suggesting the possibility of metastatic tumor. Twenty+ months after lung cancer surgery, enhanced MRI scan of the upper abdomen showed a 3.1 cm × 2.9 cm mass in the tail of the pancreas (Fig. 3). This lesion was showed slightly low signal on T1WI, mixed slightly high signal on T2WI, and had clear boundary, mild limited diffusion (the mean apparent diffusion coefficient value is approximately 1.154 × 10⁻³ mm²/s), and rim enhancement on enhanced images, which was considered as metastatic tumor. Serum tumor markers still were within the normal range.

Twenty months after lung cancer surgery, the patient was admitted to hospital for treatment of pancreatic tail lesions. At this time, the patient was in good overall condition without obvious signs and symptoms of abdominal discomfort. Imaging examinations revealed only a single metastasis of the pancreas, so “the laparoscopic extended radical resection of pancreatic cancer and splenectomy” as performed in the same month. Postoperative pathological diagnosis was keratinized squamous cell carcinoma of the tail of the pancreas (moderately to poorly differentiated) invaded the extra-adipose tissue of the pancreas, which was considered as a metastatic tumor; there was no cancer involvement at the incisal margin and no metastasis was found in 1 splenic hilar lymph node.

The patient underwent whole-body positron emission tomography-computed tomography 1 month after the operation of pancreatic metastatic tumor: the bronchial residue of the lower lobe of the right lung, the adjacent mediastinum and the right pleural lesion, the right lateral lymph node below the trachea, the liver, the serous surface of the posterior wall of the gastric fundus, and the deep surface of the left gluteus maximus lesions were all inclined to metastases. Chemotherapy was followed: gemcitabine 1.62 g D1, D8 cisplatin 40 mg D1–3, q3w, the third cycle combined with recombinant human endostatin 210 mg for 72 hours, q3w. Six months after the operation of pancreatic metastatic tumor, metastases in the mediastinal pleura, mediastinal lymph nodes, liver, bronchial stump and deep surface of the left gluteus maximus were stable, but the lesion in the posterior wall of the gastric basement was enlarged. Systemic chemotherapy and palliative radiotherapy in the upper abdomen were continued, and the overall situation was poor.

Ten months after the operation of pancreatic metastatic tumor, the patient came to the outpatient department of our hospital for “hematochezia” and received symptomatic treatment. During the telephone follow-up 11 months after the operation of pancreatic metastatic tumor, the patient’s family stated on behalf of the patient: “The patient died at the end of September 2022” (Table 1). The survival time of the patient was 30 months after lung cancer and 10+ months after lung cancer pancreatic metastasis.

**Table 1:** Timeline of events.

	Time	Postoperative lung cancer	Postoperative pancreatic metastasis	Chest CT	Abdominal CT/MRI	Head MRI	Bronchofibroscopy	Tumor markers
Preoperative lung cancer	February 2020	*	*	(+)	(−)	(−)	(+)	(−)
Lung cancer surgery	February 26, 2020		*					
Postoperative lung cancer		<12 mo	*	(−)	(−)	(−)	#	(−)
	March 2021	12+ mo	*	(+)	(−)	(−)	(+)	(−)
	May 2021	14+ mo	*	(+)	(−)/(+)	(−)	#	(−)
	July 2021	17 mo	*	(+)	(−)/(+)	(−)	#	(−)
	September 2021	19+ mo	*	(+)	(+)	(−)	#	(−)
	October 2021	20+ mo	*	(+)	(+)	(−)	#	(−)
Pancreatic metastasis surgery	November 4, 2021	20+ mo						
Postoperative pancreatic metastasis	December 2021	21+ mo	1+ mo	(+)	(++)	(−)	#	(−)
Passed away	September 2022	30+ mo	10+ mo					

* = no surgery, (+) = tumor indicated, (++) = multiple-organ metastasis, (−) = no tumor, (−)/(+) = no tumor in report, but tumor found on retrospective review, # = no relevant test done.

CT = computed tomography, MRI = magnetic resonance imaging.

## 3. Discussion

Pancreatic metastases are rare. Renal carcinomas are the most common primary tumors that metastasized to the pancreas in Europe and the United States, but in China, lung cancer is the most common primary tumors.^[13,14]^ Lung cancer is usually metastasized to adrenal gland, liver, bone and brain through blood flow,^[15,16]^ while pancreatic metastasis of lung cancer is quite rare, accounting for about 1% in live patients and 10% in cadaver biopsy.^[2]^ Maeno et al^[17]^ included 850 patients with lung cancer, and only 3% (26/850) had pancreatic metastasis. Among the pathological types of primary lung cancer, small cell lung cancer is the most commonly transferred to the pancreas, followed by adenocarcinoma and large cell carcinoma, and finally squamous cell carcinoma.^[3-5]^ Jianchun Duan et al^[18]^ showed that among 35 patients with pancreatic metastasis of lung cancer, 80% were small cell lung cancer, 8.6% adenocarcinoma, and 11.4% were squamous cell carcinoma. In the clinical analysis of 42 cases of lung cancer with pancreatic metastasis conducted by Yu Zhang et al,^[19]^ small cell lung cancer accounted for 43%, while squamous cell carcinoma only accounted for 9.5%.

Most patients with pancreatic metastases have hidden onset without obvious or specific symptoms and signs,^[20]^ and the levels of tumor markers such as CA19-9 are mostly within the normal range. Therefore, pancreatic metastases are not easy to be detected and diagnosed early. Pancreatic metastases are usually found in regular imaging examinations, and their mainly manifestations included single nodules, multiple nodules and diffuse enlargement of pancreas, among which single nodules are the most common, accounting for 50% to 70%.^[17]^

The single nodule of typical pancreatic metastasis was mostly round or oval with clear edges. CT plain scan showed equal/slightly low-density lesions, enhanced scan showed mild enhancement or uneven enhancement. On MRI images, the lesions showed slightly low signal on T1WI and slightly high signal on T2WI. The enhanced scan showed mild enhancement, or annular or uneven enhancement. Pancreatic metastases in this patient were detected in the postoperative follow-up. The lesion of the case showed oval iso-density nodules with clear edges and mild enhancement on CT images, and slightly low signal on T1WI and mixed slightly high signal on T2WI with clear boundaries and annular enhancement on MRI images, which were typical imaging manifestations of single pancreatic metastases.

In the retrospective observation of images, an iso-density nodule on abdominal enhanced CT was found, but missed report at the time of 14+ and 16+ months after lung cancer surgery. Imaging diagnosis of pancreatic metastasis was made 20+ months after operation of lung cancer. The author speculated that the patient had no symptoms and signs of abdominal discomfort at that time, and tumor markers were also within the normal range, so that careless writing and reviewing of reports can lead to missed diagnoses.

When patients with lung cancer develop pancreatic metastases, tumors are defined as stage IV, and the prognosis of the patients are generally poor.^[18,19]^ The median survival time of patients with lung squamous cell carcinoma is about 30% shorter than that of other non-small cell lung cancer subtypes.^[21]^ Nearly 50% of patients with non-small cell lung cancer have developed distant metastases when they are first diagnosed. Palliative treatment is usually adopted, and the prognosis is poor, with the 5-year survival rate as low as 4.9%. However, some scholars believe that resection of pancreatic metastases can significantly improve the prognosis of some specific patients when the primary tumor is under control. Kageyama et al^[22]^ reported that patient survived for >5 years after resection of pancreatic metastasis. Among 32 patients with pancreatic metastases of lung cancer studied by DeLuzio et al,^[7]^ the median survival time of patients who received radical surgery was 29 months, and the 2-year and 5-year survival rates were 65% and 21%, respectively. The median survival time of patients receiving palliative surgery or drug therapy was only 8 months, and the 2-year and 5-year survival rates were 25% and 8%, respectively, indicating that radical surgery is benefit to improve the survival rate. Surgical resection of pancreatic metastases can prolong survival compared with conservative treatment, and the prognosis of radical surgery is better than that of palliative surgery.^[23,24]^ This case was diagnosed with lung squamous cell carcinoma, no distant metastasis was found simultaneously, and a single pancreatic metastasis occurred 14 months after radical lung cancer resection, so radical surgical resection of pancreatic metastasis was performed. One month after the operation of pancreatic metastasis multiple organs metastases appeared, and 10 months after the pancreas operation, the patient had deceased. The survival time of the patient after lung cancer pancreatic metastasis was 10 months, much <29 months reported in the previous literature.

## 4. Conclusion

In summary, pulmonary squamous cell carcinoma with metachronous pancreatic metastasis is extremely rare. Detecting and resecting solitary pancreatic metastases promptly may prolong patient survival. In this case, pancreatic metastasis surgery occurred about 6 months after the initial imaging. At that time, no clinical signs of tumor metastasis were present, and imaging failed to detect the pancreatic lesion. Despite radical pancreatic surgery, systemic chemotherapy, and palliative radiotherapy, the patient survived only 10 months. This highlights the need for radiologists to carefully analyze images of cancer patients to identify small or occult lesions and prevent missed diagnoses. It also reminds clinicians that such patients may have occult metastases not detected by imaging. They should assess the patient’s overall condition, NER, CXI, etc, to identify high-risk patients and extend survival. Finally, the prognosis and optimal treatment for solitary pancreatic metastasis from lung cancer remain unclear, warranting more research attention.

## Acknowledgments

The authors would like to thank the patient for agreeing to publication of this report.

## Author contributions

**Data curation:** Congying Hu, Zhirong Shen, Hongen Zhen, Dongmei Rao.

**Investigation:** Hongen Zhen.

**Resources:** Congying Hu, Zhirong Shen, Jianqun Yu.

**Supervision:** Xuejin Sun, Jianqun Yu.

**Validation:** Dongmei Rao.

**Visualization:** Hongen Zhen.

**Writing – original draft:** Congying Hu.

**Writing – review & editing:** Xuejin Sun, Jianqun Yu.
